# Editorial for Special Issue: Advanced Materials and Technologies in Nanogenerators

**DOI:** 10.3390/nano12203606

**Published:** 2022-10-14

**Authors:** Zhen Wen, Hengyu Guo, Longfei Wang

**Affiliations:** 1 Institute of Functional Nano and Soft Materials (FUNSOM), Soochow University, Suzhou 215123, China; 2 Department of Physics, Chongqing University, Chongqing 401331, China; 3 Beijing Institute of Nanoenergy and Nanosystems, Chinese Academy of Sciences, Beijing 101400, China

Nanogenerators, based on Maxwell’s displacement current as the driving force, have inspired a new and developing field since their invention in 2006. In less than 20 years, nanogenerators have rapidly developed into a research hotspot in the field of energy harvesting and self-powered sensing. Based on new technologies and nanomaterials, nanogenerators can efficiently harvest and store energy from the environment for the sustainable operation of micro systems. This lays the foundation for the popularization of their broad applications in energy science, environmental protection, wearable electronics, self-powered sensors, medical science, robotics, and artificial intelligence. The Special Issue ‘Advanced Materials and Technologies in Nanogenerators’ selected 12 articles, including 9 original research articles and 3 reviews, to show the current applications and future evolution of nanogenerators.

Marcos Duque and Gonzalo Murillo [1] reported a triboelectric nanogenerator (TENG) that can harvest mechanical energy by manual tapping. In order to create a high-performance TENG, the authors improved the charge density by correctly selecting materials, designing new spacers, and charging high-voltage corona for charge injection. This kind of TENG can provide a sustainable power supply for miniature electronic sensors.

Yuan et al. [2] proposed a high-performance coniform Helmholtz resonator-based TENG for efficiently harvesting acoustic energy. The novel design in the CHR-TENG can improve its output performance and broaden its response band over harvesting acoustic energy. With the optimized design, the maximum acoustic sensitivity per unit area of the CHR-TENG can reach 1.68 V/Pa·m^2^, while the power density per unit of sound pressure is 2.88 W/Pa·m^2^, obtaining a 58.2% improvement over previously reported results. In addition, the CHR-TENG was demonstrated to charge a 1000 µF capacitor up to 3 V in 165 s, power a sensor for continuous temperature and humidity monitoring, and light up as many as 464 commercial LED bulbs for acoustic energy harvesting.

Shi et al. [3] described a system that activates O_2_ via the direct modulation of its spin state by mechanical energy-induced triboelectric corona plasma, enabling a CO oxidation reaction under normal temperature and pressure. Under optimized reaction conditions, the activity was 7.2 µmol h^−1^, and the energy consumption per mole CO was 4.2 MJ. The results of kinetic isotope effect, colorimetry, and density functional theory calculation studies demonstrated that electrons generated in the triboelectric plasma were directly injected into the antibonding orbital of O_2_ to form highly reactive negative O_2_^−^ ions, which effectively promoted the rate-limiting step of O_2_ dissociation. The barrier of the reaction of O_2_^−^ ions and molecular CO was 3.4 eV lower than that of O_2_ and molecular CO. This work provides an effective strategy for using renewable and green mechanical energy to realize spin-forbidden reactions of small molecules.

Wan et al. [4] constructed a self-powered magnetic sensor based on a subtle triboelectric nanogenerator that consists of a magnetorheological elastomer (MRE). This magnetic sensor relies on triboelectrification and electrostatic induction to produce electrical signals in response to the MRE’s deformation induced by the variational magnetic field without using any external power sources. The fabricated magnetic sensor showed a fast response of 80 ms and a desirable sensitivity of 31.6 mV/mT in a magnetic field range of 35–60 mT as well as preliminary vectorability enabled by the multichannel layout. Their work provides a new route for monitoring dynamic magnetic fields and paves the way for self-powered electric–magnetic coupled applications.

Zhang et al. [5] proposed a triboelectric technology-based all-in-one self-powered HMI (Human–Machine Interaction) system. The hydrogel-based triboelectric nanogenerator harvests mechanical energy from the human body and provides power for the whole system. Gesture sensing signals are wirelessly transmitted to the intelligent car for remote telemetry and control. This technology expands the application potential of wearable devices integrated in self-powered HMI systems.

Ding et al. [6] reported a new triboelectric nanogenerator based on sodium chloride powder (S-TENG) to obtain mechanical energy. The polytetrafluoroethylene (PTFE) film and sodium chloride powder layer serve as the triboelectric pair. After testing and calculation, the internal resistance of S-TENG was 30 MΩ, and the output power of S-TENG could arrive at about 403.3 µW. Furthermore, the S-TENG can achieve an open-circuit voltage (Voc) of 198 V and a short-circuit current (Isc) of 6.66 µA, respectively. Moreover, owing to the moisture absorption of sodium chloride powder, the S-TENG device also has the function of a humidity sensor. This work proposed a functional TENG device, and it can promote the advancement of self-powered sensors based on the TENG devices.

Wang et al. [7] introduced a highly conductive organogel ion conductor that acts as a self-powered sensor. The addition of small-molecular-weight lithium salt resulted in a conductivity of up to 9.1 × 10^−4^ S·cm^−1^ and the organogel exhibited excellent temperature stability in the temperature range of −70 to 100 °C. This gel-based piezoelectric sensor provides a stable energy output under pressure and can withstand long-term durability tests.

Liu et al. [8] reported a framework for monitoring the evolutionary path of nanogenerator technology based on the Sentence-BERT and phrase-mining methods. The method combines both scientific and technological dimensions to analyze technological evolution in the field of nanogenerators through an improved theme-evolution modeling approach (SKT) with multi-source text vectorization.

Liu et al. [9] proposed a novel framework to monitor the evolutionary pathways of nanogenerator technology based on multi-source data and a knowledge graph. In the framework, the knowledge graph makes full use of text information, and the multi-source data fully considers the evolutionary pathways from different data perspectives. While analyzing the evolution pathways of some developed nanogenerator technologies, this research finds several emerging research directions for nanogenerators, such as novel energy sources and fiber structure of nanogenerators.

Li et al. [10] reviewed the recent research progress of rare earth metal-modified metal halide perovskite materials and their corresponding optoelectronic devices. First of all, Ln^3+^ ions made up the deficiency of utilization for solar spectrum by perovskite materials. In addition, Ln^3+^ ions were used as dopants to improve device performance. Finally, Ln^3+^ ions were also used in the fields of photodetectors and solar luminescent concentrators. It indicates the huge potential of rare-earth metals in improving the performances of the perovskite optoelectronic devices.

The review authored by Cao et al. [11] discussed the latest technical issues and prospects of TENG in fluid dynamics sensing. The authors introduced the advantages and key problems of TENG applied in fluid dynamics sensing, which has attracted more scholars’ attention. This review analyzed the principles of the TENG and illustrated the feasibility and advantages of using the TENG as a fluid dynamics sensor for local fluid phenomena and environments. The authors summarized the recent works of the TENG as a fluid dynamics sensor and help guide the future direction of the TENG in fluid dynamics sensing.

Si et al. [12] summarized the working mode and basic theory of TENGs. Then, the authors reviewed the applications of TENGs in AR, VR, and other wearable electronic devices. In addition, this paper also summarized the design methods of TENGs as self-powered sensor modules in these devices. Finally, the authors analyzed and proposed future application areas.

In summary, this Special Issue discusses the wide applications, new materials, and evolution in the field of nanogenerators. I hope these articles will be beneficial for readers in future research and more new creations will be submitted to this journal.
